# Imaging Advances in Bronchoscopy Through the Combination of High‐Definition Bronchoscopes and New Image Processing Systems

**DOI:** 10.1111/1759-7714.70065

**Published:** 2025-04-11

**Authors:** Kei Morikawa, Hirotaka Kida, Hiroshi Handa, Masamichi Mineshita

**Affiliations:** ^1^ Department of Respiratory Diseases St. Marianna University School of Medicine Kawasaki Japan

**Keywords:** high‐definition (HD) bronchoscope, red dichromatic imaging (RDI), texture and color enhancement imaging (TXI)

## Abstract

Bronchoscopy has become crucial with advances in diagnosing and treating lung cancer and interstitial pneumonia. Integrating bronchoscopes with endobronchial ultrasonography and navigation systems has significantly improved diagnostic yields and procedural safety. Although improvements in image quality and bronchoscope operability are evident, these benefits are often recognized based on widespread usage rather than objective assessment. In this study, we aimed to compare how the appearance of lesions changes depending on new modes on a case‐based images, with white light observation as a standard image. High‐definition bronchoscopes have introduced notable clinical advantages, enhancing direct visualization of lesions and enabling precise evaluation of histological changes. This enhanced visualization facilitates better detection of vascular patterns, which can improve safety during transbronchial biopsy by minimizing complications. The addition of the CV‐1500 image processor allows for comprehensive image enhancement, such as brightening peripheral bronchi, emphasizing the structural presence of lesions and blood vessels, and estimating blood vessel depth through color tone variations reflecting absorbance differences. The combination of high‐definition bronchoscopy and advanced image processing holds promise for accumulating more robust clinical knowledge and optimizing diagnostic strategies.

## Graphical abstract

High‐definition bronchoscopes and new image processing technology allow us to obtain bronchoscopic images of unprecedented high quality. In bright images with BAI‐MAC support, subepithelial blood vessels and their depths are clearly visible using TXI and RDI modes, making it possible to perform procedures based on objective indicators.

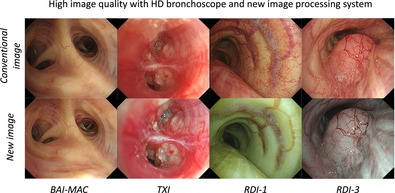

## 1 Introduction

The performance of bronchoscopes has advanced significantly over time, with image quality and operability remaining the most critical indicators of innovation. Image resolution has evolved, moving from the earliest generation of fiberscopes to the attachment of charge‐coupled device (CCD) cameras, transitioning through hybrid scopes, and culminating in electronic scopes, which have become mainstream. Additionally, various observation modes have been developed, reconstructing electronic information through different processing techniques [1, 2].

In recent years, combining high‐definition bronchoscopy with new image processing systems has elevated bronchoscopy image quality to an unprecedented level, comparable to that of gastrointestinal endoscopy [3, 4]. This enhancement contributes not only to detailed lesion observation and assessment of spread and depth but also improves procedural safety. In particular, it is unclear for what kind of lesions the new system is useful. Hence, the purpose of this study is to collect and compare case‐based images of how bronchoscopic views change in different new modes, with white light observation as a standard image. This article explores the functions and clinical value of the different observation modes enabled by the combination of high‐definition bronchoscopy and the CV‐1500, a development poised to become an irreversible standard in clinical practice.

## 2 Methods

### 2.1 BF‐H1200, BF‐1TH1200: 1200 Series Bronchoscopes

**FIGURE 1:**
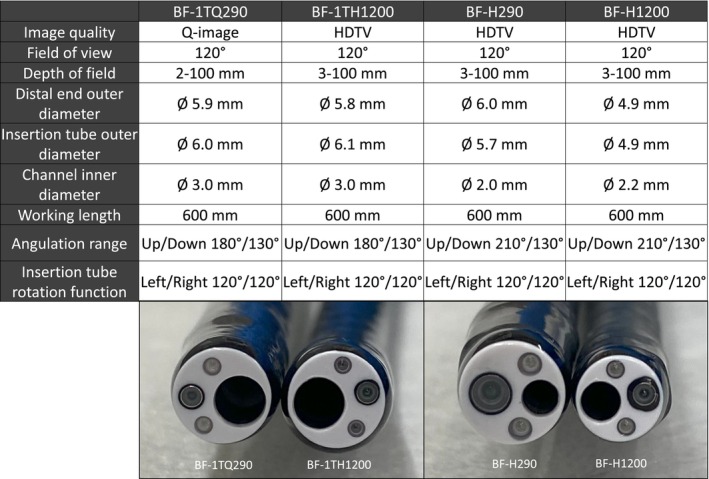
Differences in specifications between conventional and new bronchoscopes.

The BF‐H1200 is a diagnostic bronchoscope, and the BF‐1TH1200 is a therapeutic bronchoscope (Olympus Medical Systems, Tokyo, Japan) (Figure 1). Both models are equipped with high‐sensitivity CMOS image sensors and are compatible with the CV‐1500. The BF‐H1200 features a smaller distal end outer diameter (4.9 mm) with a larger channel inner diameter (2.2 mm working channel) than its predecessor, the BF‐H290 (2.0 mm working channel). The BF‐1TH1200 provides HD image quality and has a smaller distal end outer diameter (5.8 mm) than its predecessor, the BF‐1TQ290 (5.9 mm) while maintaining the same channel inner diameter (3.0 mm working channel).

### 2.2 CV‐1500: EVIS X1 Video System Center

CV‐1500 is newly equipped with violet/blue/green/amber/red five‐color LEDs. By employing a set of five‐color LEDs, it becomes feasible to encompass the full spectrum of wavelengths present in conventional xenon light sources. The newly adopted amber LED especially improves red color reproduction in white light imaging (WLI) and also allows the implementation of a new image enhancement endoscopy (IEE) technology, Red dichromatic imaging (RDI), in addition to the conventional IEEs such as NBI. The texture and color enhancement function (TXI) is also newly incorporated in CV‐1500. These IEEs will be elaborated on subsequently.

### 2.3 Description of Various Modes and Case Presentation

#### 2.3.1 Brightness Adjustment Imaging With Maintenance of Contrast (BAI‐MAC)

**FIGURE 2:**
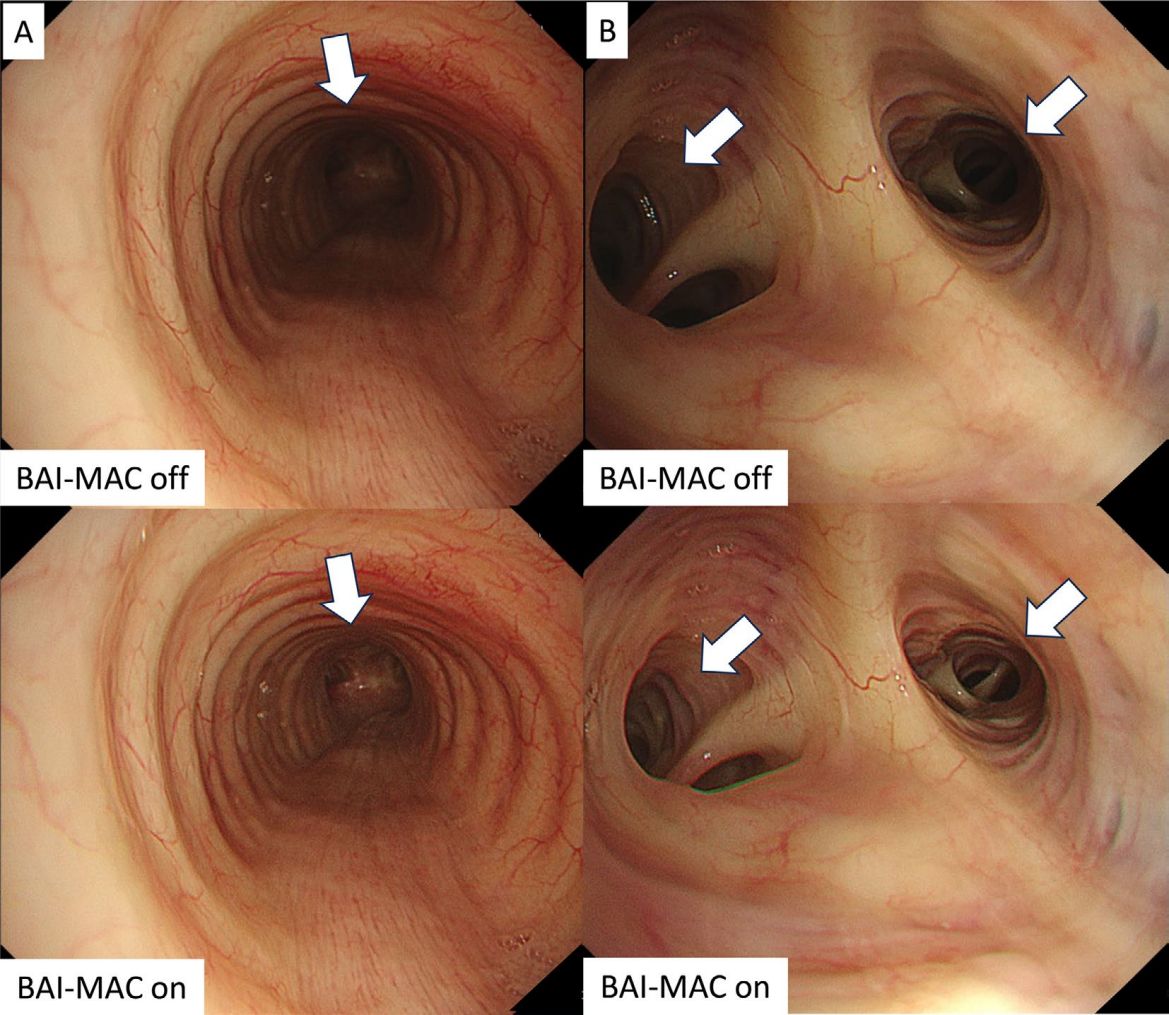
Differences in image brightness due to BAI‐MAC function in the trachea (A) and peripheral bronchi (B). BAI‐MAC, brightness adjustment imaging with the maintenance of contrast.

Most bronchoscopic images are captured in the long axis of the bronchi, encompassing both proximal and distal areas within a single field of view, necessitating adjustments in brightness and focus. The BAI‐MAC function enhances the brightness of the distal area while maintaining the brightness of the proximal area without causing halation. This enhancement is achieved through image processing that separates brightness and texture analysis, correcting darker areas and blending image information automatically. With the BAI‐MAC function, the tracheal carina can be seen clearly when viewed from above the trachea (Figure 2A). Additionally, the improved visualization of peripheral bronchial branches facilitates selecting the correct access bronchus (Figure 2B). This function acts like a guiding light that naturally illuminates the next step to take.

#### 2.3.2 Texture and Color Enhancement Imaging (TXI)

TXI is a function designed to emphasize image information by combining the three image processing algorithms of brightness correction of the dark part of the image without halation, color difference expansion processing, and texture component emphasis processing. In the TXI algorithm, the original input image is first separated into a detail layer and a base layer. In the digestive field, TXI is used for the detection or diagnosis of early gastric cancer and the evaluation of the healing process of inflammatory bowel disease [5, 6, 7].

**FIGURE 3:**
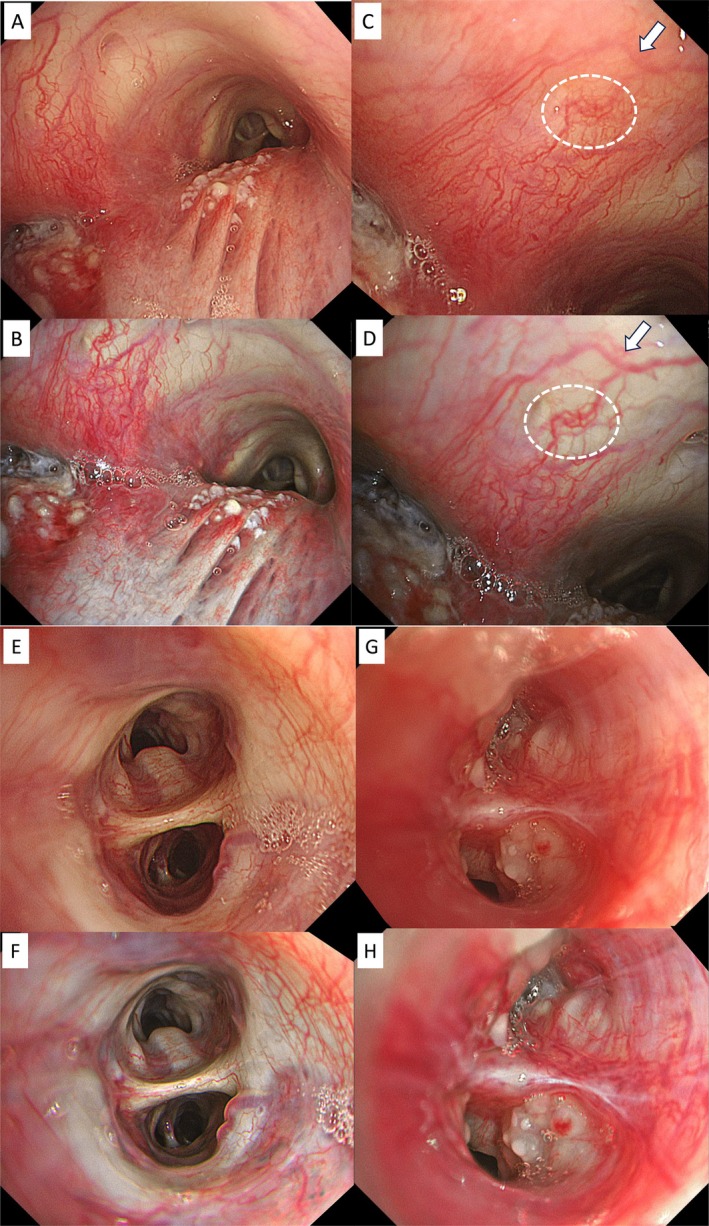
Tracheal invasion of esophagus cancer (B), close‐up images of the same case (D), epithelialized blood vessels (F), and peripheral lesion (H) in bronchi clearly shown in structure‐weighted images by TXI, compared with white light images (A, C, E, G, respectively). TXI, texture and color enhancement imaging.

For bronchial field, structural enhancement, for instance, provides stronger contrast to the longitudinal folds of the membranous region, improving the visibility of subepithelial blood vessels. In the case of left bronchial stenosis due to esophageal cancer invading the left bronchus, TXI facilitates distinguishing various findings such as epithelial redness, edema, and skip lesions with local necrosis (Figure 3A,B). Subepithelial blood vessels can often be recognized more three‐dimensionally (Figure 3C–F), allowing depth estimation and distribution more clearly. Especially, close‐up images of Figure 3A,B case showed the blood vessels are easily distinguishable particularly around the white arrow and white dotted line in comparison with the white light image (Figure 3C,D).

With thin high‐definition scopes (BF‐H1200), the ability to capture detailed visual findings of peripheral lesions has increased. Slight discoloration of the subepithelial layer to a white tone aids in distinguishing blood vessels and enhances peripheral brightness without increasing halation (Figure 3G,H). TXI has two modes: TXI‐1 and TXI‐2, with TXI‐2 providing a color tone closer to white light.

#### 2.3.3 Red Dichromatic Imaging (RDI)

RDI is an IEE that is based on narrow‐band light with long wavelengths. Short wavelengths of light, which are utilized in narrow‐band imaging (NBI) technology, are absorbed by capillaries on the mucosal surface and veins in the submucosa, whereas the long wavelengths of light penetrate more deeply into the mucosa. Hence, RDI supports visualization of bleeding points and deep blood vessels.

**FIGURE 4:**
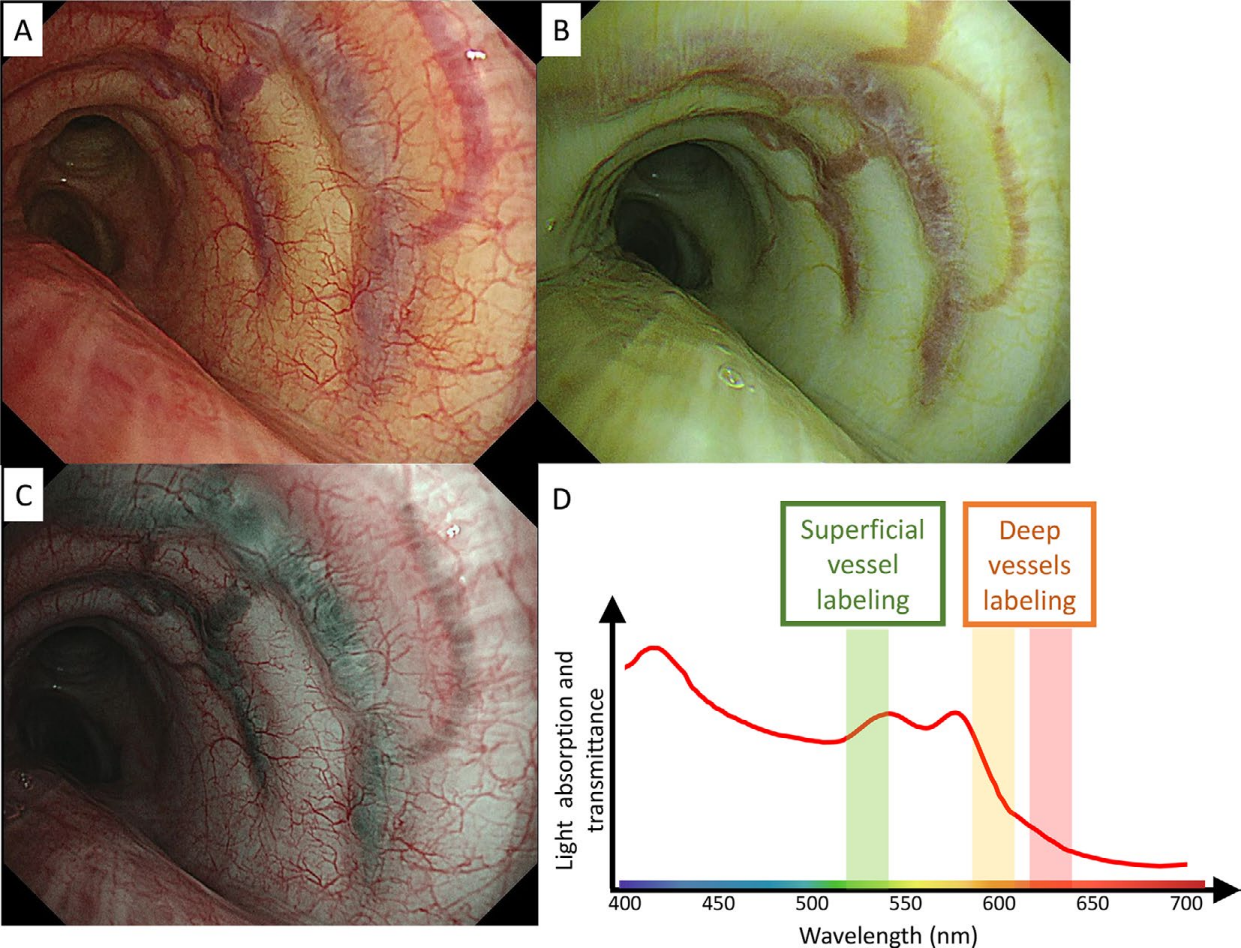
Evaluation of subepithelial vessels of the left main bronchus using WLI (A), RDI‐1 (B), RDI‐3 (C), and light wavelength and absorbance distribution (D). RDI, red dichromatic imaging; WLI, white light imaging.

RDI‐1 and RDI‐2 are modes where superficial blood vessels appear yellow to orange and deep vessels appear dark orange to red. This occurs because short‐wavelength colors are reflected from the surface, while long‐wavelength colors penetrate and reflect from deeper areas (Figure 4A,B). The main distinction is that RDI‐2 has a darker color tone than RDI‐1. Conversely, RDI‐3 reverses this color scheme, displaying deep vessels in green and superficial ones in red, with particularly high visibility of deep vessels (Figure 4C). Deep vessels often have a large diameter and high blood flow rate, making them critical to avoid during biopsy.

**FIGURE 5:**
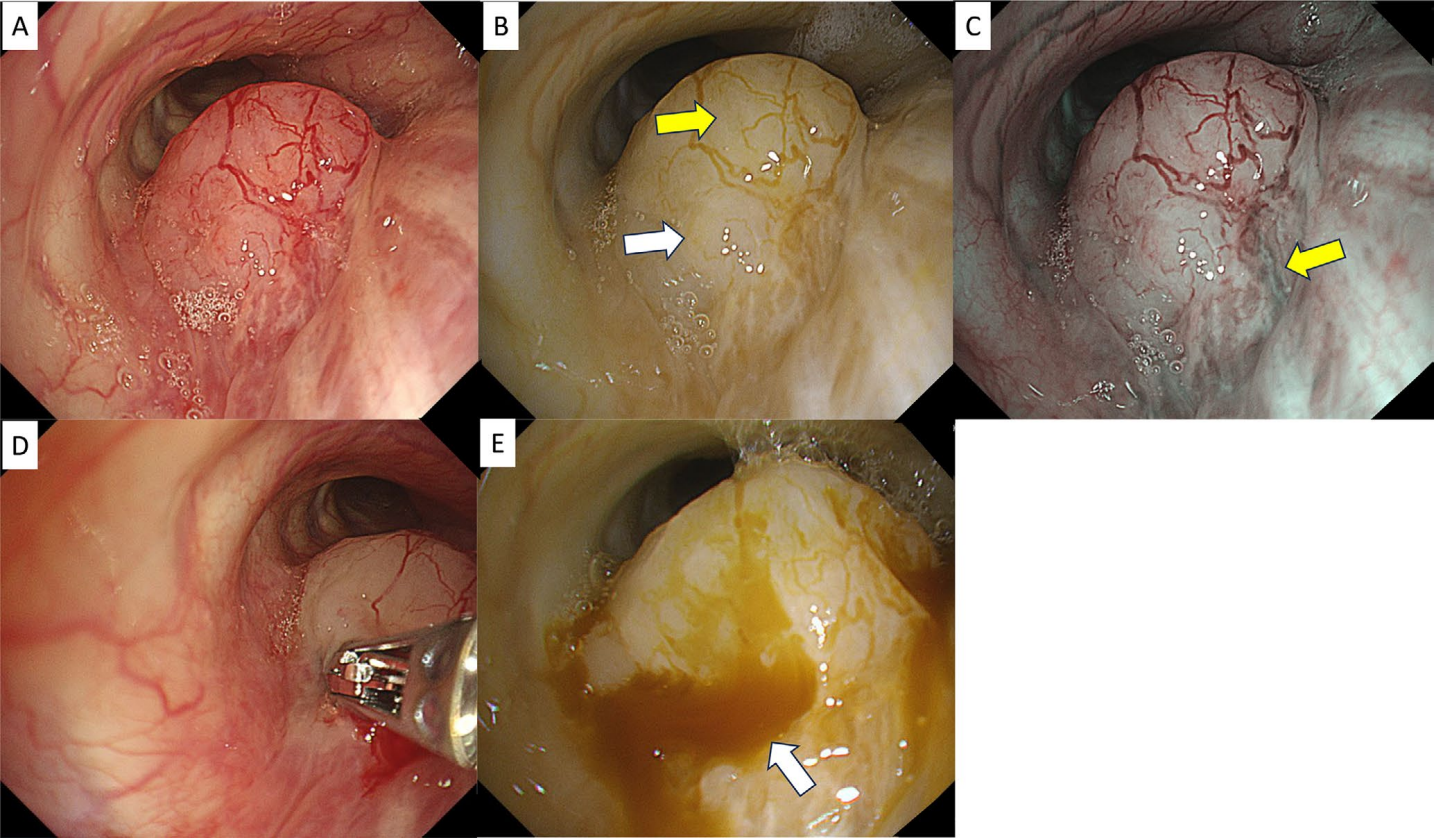
Observation using WLI (A), RDI‐1 (B), and RDI‐3 (C) for tumors in the right middle trunk, biopsy scene (D), and observation of biopsy site using RDI‐1 (E). RDI, red dichromatic imaging; WLI, white light imaging.

Initially developed for the gastrointestinal field for procedures such as confirming deep blood vessels in endoscopic submucosal dissection (ESD) and peroral endoscopic myotomy (POEM) and identifying bleeding points [8, 9, 10], RDI is also expected to be beneficial in bronchoscopy. When biopsying a tumor, RDI gives detailed information about blood flow, facilitating site selection to minimize bleeding. The color tone can also indicate local blood flow. For example, a tumor in the right intermediate trunk (Figure 5A) appears slightly yellow on the ventral side in RDI‐1 (Figure 5B, yellow arrow) and slightly whitish in the center (Figure 5B, white arrow). RDI‐3, however, reveals deep vessels as green streaks running from the membranous portion to the tumor (Figure 5C, yellow arrow), indicating caution is needed. In such cases, a biopsy can first be performed from the center, with subsequent checks of the degree and bleeding point using RDI‐1 (Figure 5D,E).

## 3 Discussion

Recent advances in respiratory endoscopy have shown improved diagnostic rates due to additional techniques or devices [11, 12, 13], but few reports have focused on enhanced image quality or functional diversification [14, 15]. Conversely, the diagnostic accuracy of gastrointestinal endoscopy has benefited from combining direct visualization with artificial intelligence (AI), leading to the release of devices equipped with this function [16]. Differential diagnosis based on direct bronchoscopy findings is well established in clinical practice, using image findings to assess histological type, invasion depth, and benign or malignant nature for guiding diagnosis and treatment. Considering the future spread of AI, it will be necessary for bronchoscopy images to be of high image quality, as differential diagnosis will become easier through deep learning [17].

Improved image quality is expected to enhance diagnostic accuracy and provide more detailed information on pathological changes. In the cases presented, recorded with high‐definition bronchoscopy, the information regarding the pathology of lesions and their surroundings has significantly increased. The most critical aspect of bronchoscopy for diagnosing malignant tumors is ensuring safety while obtaining viable cancer cells. The most common and troublesome event affecting procedural safety is bleeding [18]. Traditionally, superficial blood vessel characteristics and courses were assessed with NBI, but TXI offers a more precise contrast that can estimate the depth of blood vessels. RDI is even more objective, confirming vessel depth through color tones. By utilizing these modes, selecting safe biopsy sites based on objective information has become possible. RDI is also expected to identify bleeding points when bleeding occurs [9].

Lesions with minimal blood flow often exhibit necrotic changes. While biopsy of such lesions is safer due to the reduced risk of bleeding, it may not aid in cancer diagnosis. Checking blood flow to identify necrotic areas using RDI or NBI is crucial. Balancing the need to avoid bleeding while obtaining viable cancer cells is challenging; failure to do so can result in heavy bleeding or missed cancer diagnosis. Identifying the best biopsy site that aligns with both goals is essential, and high‐definition bronchoscopes with advanced image processing technology are expected to address these challenges in clinical practice. Detailed and objective image evaluation has the potential to improve diagnostic rates, avoid complications, and ultimately shorten examination times. By linking high image quality and AI, there is also the potential to establish new diagnostic methods.

The first limitation of this study is that it is limited to five representative cases demonstrating the usefulness of high‐definition image quality at a single institution. Second, the number of lesions observed under direct vision was limited, and imaging with various modes was not possible in all cases undergoing bronchoscopy due to the effects of bleeding and sedation. On the other hand, the time required to capture images in each mode was within a few minutes, and there was no concern about an increase in complications. Finally, regarding potential bias from the endoscopic device development company, as described in a footnote, they had no involvement in the study design and interpretation.

## Author Contributions

K.M. had full access to data in this perspective article and takes responsibility for the integrity and accuracy of data analysis. K.M., H.K., H.H., and M.M. contributed to bronchoscopic examination and interpretation. K.M., H.K., H.H., and M.M. contributed to the scientific review and final approval of this manuscript. All authors read and approved the final manuscript.

## Ethics Statement

Written informed consent was obtained from the patient for publication of this case presentation and any accompanying images. Image acquisition was with ethics approval (OLET‐2022‐004).

## Conflicts of Interest

K.M. has received research honoraria from Olympus Corporation. All other authors declare no conflicts of interest.

## Data Availability

Data are provided within the manuscript, but other images are available upon request.
